# The Link Between 15-F_2t_-Isoprostane Activity and Acute Bovine Endothelial Inflammation Remains Elusive

**DOI:** 10.3389/fvets.2022.873544

**Published:** 2022-04-29

**Authors:** Ashley K. Putman, Lorraine M. Sordillo, G. Andres Contreras

**Affiliations:** Department of Large Animal Clinical Sciences, College of Veterinary Medicine, Michigan State University, East Lansing, MI, United States

**Keywords:** isoprostane, inflammation, oxidative stress, dairy cattle, endothelial cell

## Abstract

Modern dairy cattle suffer from increased incidence and severity of mastitis during major physiological transitions of the lactation cycle. Oxidative stress, a condition resulting from inadequate antioxidant defense against reactive oxygen and nitrogen species, is a major underlying component of mastitis pathophysiology. Isoprostanes (IsoP) are molecules derived from cellular lipid membranes upon non-enzymatic interaction with reactive species during inflammation, and are regarded as highly sensitive and specific biomarkers of oxidative stress. Changes in IsoP concentrations have been noted during major physiological transitions and diseases such as coliform mastitis in dairy cattle. However, the biological role of IsoP during oxidative stress in dairy cows has not been well-elucidated. Therefore, this study aimed to characterize the impacts of IsoP on oxidative stress outcomes in a bovine model of acute endothelial inflammation. Bovine aortic endothelial cells (BAEC; *n* = 4) were stimulated with 2,2'-azobis (2-amidinopropane) dihydrochloride (AAPH) or lipopolysaccharide (LPS) with or without 15-F_2t_-IsoP to determine how IsoP influence oxidative stress outcomes. Our endothelial inflammation model showed relatively decreased reactive metabolites and increased barrier integrity in cells treated with both the agonist and IsoP compared to agonist treatment alone. However, IsoP do not appear to affect oxidative stress outcomes during acute inflammation. Understanding the effect of IsoP on BAEC is an early step in elucidating how IsoP impact dairy cows during times of oxidative stress in the context of acute clinical mastitis. Future studies should define the optimal dosing and treatment timing of IsoP to maximize their cytoprotective potential during acute inflammation.

## Introduction

Modern dairy cows are subject to increased incidence and severity of mastitis during major physiological transitions of the lactation cycle. Principal underlying components of mastitis pathophysiology include dysregulated inflammation and oxidative stress resulting from the perturbations in immune system function and redox balance during these transitions (1, 2). For instance, increased energy demands to support copious milk production after calving necessitate greater reactive oxygen and nitrogen species (RONS) production from the mitochondria. At the same time, decreased feed intake leads to insufficient consumption of antioxidants that defend against RONS (2). Such disruptions to redox balance can lead to oxidative stress, which occurs when proteins, nucleic acids, and lipids are damaged as a result of RONS overwhelming host antioxidant defenses (3). Severe cellular damage from increased RONS during oxidative stress can lead to numerous consequences, including endothelial dysfunction.

A functional endothelium is crucial for appropriate inflammatory responses. Endothelial cells (EC) form a single cell layer between blood and tissue and thus, are responsible for activities such as maintaining vascular barrier integrity (4). Dysfunctional EC allow for sustained dysregulated inflammation, leading to impaired immunity and increased disease susceptibility. For instance, apoptosis and necrosis are common features of dysfunctional EC (5). Decreased barrier integrity permits increased flux of activated leukocytes and fluid into tissues, perpetuating inflammation (6). Certain lipid mediators formed during inflammation, known as oxylipids, can induce EC dysfunction. For example, 13-HPODE is associated with apoptosis and subsequent necrosis of bovine mammary EC along with decreased barrier integrity (7). In fact, dysregulated inflammation and the associated altered barrier integrity is a key pathological finding in coliform mastitis (8). As EC are particularly susceptible to RONS attack, they will form specialized oxylipids known as isoprostanes (IsoP) during acute inflammation (9, 10).

Isoprostanes are generated non-enzymatically when RONS react with polyunsaturated fatty acids in lipid membranes (9). Although numerous biomarkers of oxidative stress exist, IsoP are currently considered superior due to their highly sensitive and specific nature in detecting lipid peroxidation (11). At present, the omega-6-derived 15-F_2t_-IsoP is the most commonly referenced IsoP in the literature (12). Indeed, growing evidence supports plasma IsoP as a biomarker of oxidative stress in dairy cattle, including for diseases such as coliform mastitis (13–15). Isoprostanes may have a role beyond being biomarkers as several studies have established that IsoP have bioactivity on the vasculature, primarily by constricting or dilating blood vessels (16, 17). Furthermore, IsoP demonstrate an ability to alter inflammatory gene regulation, affecting cytokine production and macrophage adhesion to the vasculature (18–20). However, the physiological role of IsoP in the context of acute inflammation in dairy cattle remains poorly characterized. Thus, this study aimed to determine how IsoP alter oxidative stress outcomes in bovine endothelial cells under inflammatory challenge.

## Materials and Methods

### Reagents

Fetal bovine serum was provided by Hyclone Laboratories, Inc. (Logan, UT). HAM's F-12K and HEPES buffer were from Corning Inc. (Corning, NY). Sodium selenite, insulin, heparin, and transferrin were from Sigma-Aldrich (St. Louis, MO). Antibiotics/antimycotics, glutamine, trypsin-EDTA, and bovine collagen were purchased from Life Technologies (Carlsbad, CA). Indomethacin, 15-F_2t_-IsoP (formerly 8-isoPGF_2α_), and 2,2'-azobis (2-amidinopropane) dihydrochloride (AAPH) were purchased from Cayman Chemical (Ann Arbor, MI). Lipopolysaccharide (LPS) purified from *E. coli* 0111:B4 was purchased from Invivogen (San Diego, CA).

### Cell Culture

Primary BAEC were used to model systemic vascular acute inflammation. The methods used to isolate BAEC have been previously described by Mavangira and coworkers (8). Isolated EC were distinguished from other cell types with von Willebrand factor staining and morphological analysis. Cells were stored in liquid nitrogen at pass 4 until being thawed out for experiments. Upon thawing cells, BAEC were grown to 75-80% confluency in flasks (Corning Inc., Corning, NY) incubated at 5% CO2 and 37°C, and were used from passages 6-9. Cells were cultured in BAEC media containing HAM's F-12K, 20 mM HEPES, 10% fetal bovine serum, 100 U/mL antibiotic/antimycotic consisting of penicillin, streptomycin, and amphotericin B, 300 mg/mL L-glutamine, 10 ng/mL sodium selenite, 10 μg/mL insulin, 100 μg/mL heparin, and 5 μg/mL transferrin.

### Experimental Design

**Figure 1:**
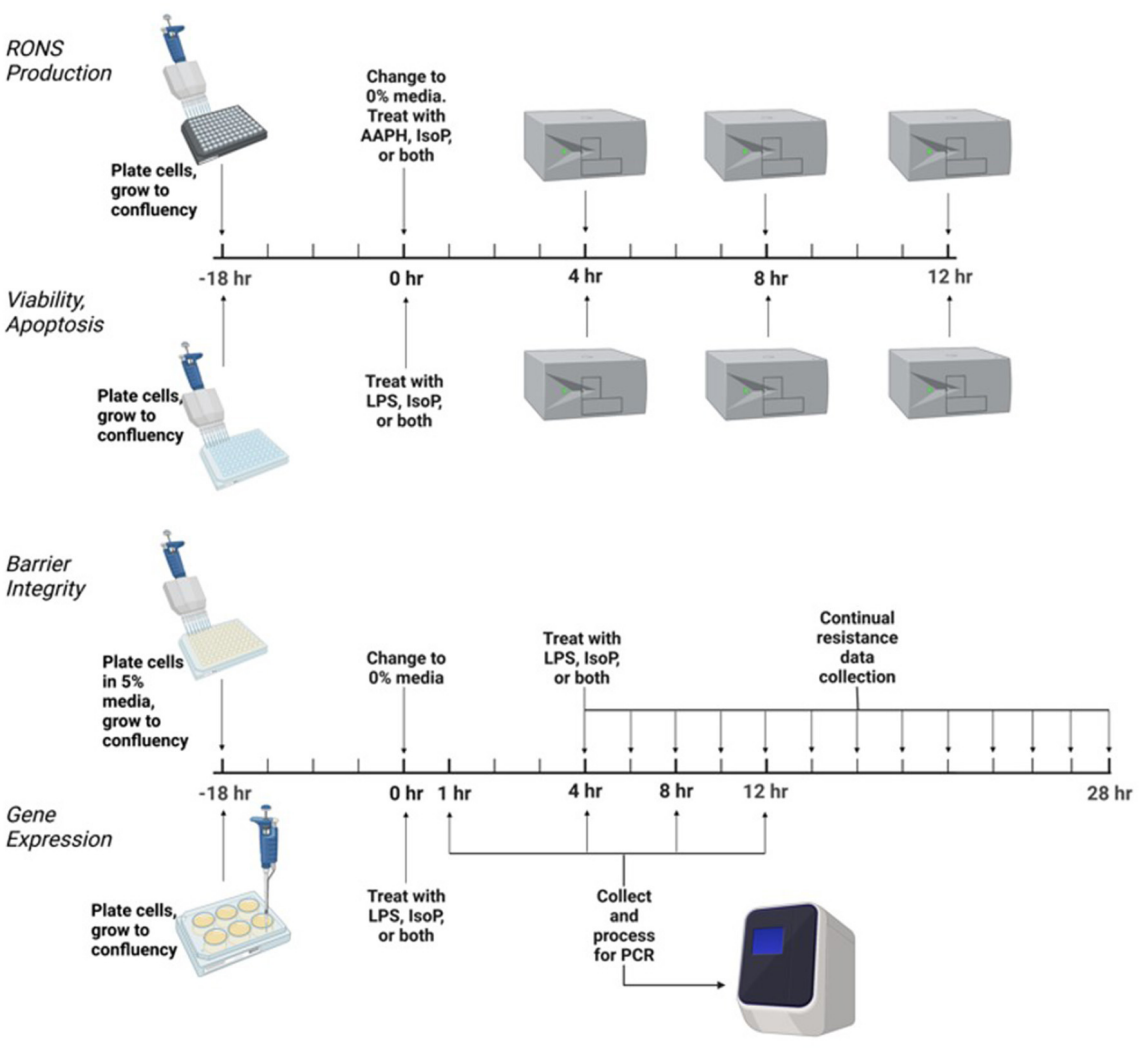
Experimental design to test the effect of isoprostane (15-F_2t_-IsoP) on bovine aortic endothelial cells (*n* = 4). AAPH, 2,2'-azobis (2-amidinopropane) dihydrochloride; LPS, lipopolysaccharide. Figure created with Biorender.com.

Conditions of increased ROS generation and acute inflammation were generated using AAPH or LPS. Treatments were as follows: untreated media control, vehicle control with 0.05% ethanol by volume, positive control of either 5 mM AAPH or LPS (15 or 25 ng/mL) as an agonist, physiologically relevant doses of 15-F_2t_-IsoP (10–500 nM), and co-culture of either AAPH or LPS with 15-F_2t_-IsoP. Incubation times varied by assay, ranging from 1 to 24 h. All BAEC treatments were delivered in F-12K media with either 10% or 0% FBS. A graphical summary of the experimental design is in Figure 1.

### Cellular Viability

Cellular viability was assessed with Promega CellTiter-Glo Luminescent Cell Viability Assay (Madison, WI). This assay produces a luminescent signal proportional to the amount of ATP generated from metabolically active cells present in a well. Cells were plated in white flat-bottom 96-well plates at a density of 4.0 × 10^4^ cells/well. The assay was then performed according to manufacturer's instructions. Luminescence was read on a Tecan Infinite 200 Pro (Männedorf, Switzerland).

### Apoptosis

To determine if IsoP were influencing apoptosis in BAEC, a Promega Caspase-Glo 3/7 kit was employed. Briefly, caspase activation of the substrate releases aminoluciferin. Subsequent interaction of the free aminoluciferin with luciferase results in a luminescent signal proportional to caspase 3/7 activity. Similar to the viability assay, cells were plated in a white flat-bottom 96-well plate at a density of 4.0 × 10^4^ cells/well. Manufacturer's instructions were followed to carry out the assay. Luminescence was read on a Tecan Infinite 200 Pro.

### RONS Production

Total intracellular RONS production was assessed with Cell Biolabs OxiSelect Intracellular RONS kit (San Diego, CA). All reactive species of the sample were measured utilizing a dichlorodihydrofluorescin DiOxyQ (DCFH-DiOxyQ) probe. In brief, RONS will interact with the highly reactive DCFH, which rapidly oxidizes to 2,7'-dichlorodihydrofluorescein, producing a fluorescent signal. Therefore, fluorescence is directly proportional to the amount of reactive species in a sample. Cells were plated at 4.0 × 10^4^ cells/well in black clear-bottom 96-well plates. After 18 hr, 10% FBS media was removed and replaced with 0% treatment media. The assay was then performed according to manufacturer's instructions. A Tecan Infinite 200 Pro was employed to read fluorescence at 480 nm excitation and 530 nm emission.

### Cell Barrier Integrity

Endothelial cell barrier integrity was assessed utilizing an electric cell-substrate impedance sensing (ECIS) ZTheta system (Applied Biophysics, Troy, NY). Cells were seeded (density = 1.0 × 10^5^) on a 96-well array (96W10idf, Applied Biophysics) in 5% FBS F-12K media and allowed to grow for ~18 h until resistances stabilized, suggesting a confluent monolayer of endothelial cells. Once confluency was reached, media was changed to 0% FBS and the resistances were allowed to equilibrate for ~4 h. Treatments as listed above in the experimental design section were added. The ECIS system took measurements every 180 s at multiple frequencies for 24 h. The resistance measurements taken at 4,000 Hz after treatment were utilized for analysis.

### Gene Expression *via* qRT-PCR

Cells were cultured in 6-well plates for RNA extraction. Wells were seeded at 1.0 × 10^6^ cells and grown to 75–80% confluency. Each well was washed twice with HBSS and then 300 μL Buffer RLT (Qiagen, Hilden, Germany) was added to lyse the cells. The buffer was collected from each tube and then added to a 1.5 mL microcentrifuge tube and cell lysate was stored at −20°C pending RNA extraction within 1 mo of collection.

Extraction of RNA occurred utilizing a Promega Maxwell RSC Instrument following the manufacturer's protocol. Quantification and the quality of RNA was assessed with a Nanodrop ND-1000 spectrophotometer (ThermoFisher, Waltham, MA) and then stored at −20°C until cDNA was generated. The A_260/280_ ratios for all samples were between 1.9 and 2.1. Prior to cDNA generation, all samples were standardized with nuclease-free water to 100 ng/μL. An equal volume of master mix containing 10 × reverse-transcription buffer, 25 × dNTP, 10 × random primers, Multiscribe reverse transcriptase, RNase inhibitor, and RNase nuclease-free water from a high-capacity cDNA reverse-transcription kit with RNase inhibitor (Applied Biosystems High Capacity cDNA Archive Kit, Waltham, MA) was added to each standardized RNA sample. Samples were placed in a MiniAmp Plus Thermal Cycler (Applied Biosystems, Waltham, MA) programmed with the following settings: 25°C for 10 min, followed by 37°C for 2 h, then 85°C for 5 min, finishing with 4°C hold until samples are removed. Samples were then stored at −20°C until qRT-PCR was completed.

Real-time PCR was carried out with predesigned TaqMan primers and FAM-MGB probes (Applied Biosystems). Genes that have demonstrated alterations in the face of IsoP challenge and are also relevant to mastitis pathophysiology were selected for analysis (20–22). Samples were assessed for nitric oxide synthase 2 (*NOS2*, Bt03249586_m1), peroxisome proliferator activated receptor alpha (*PPARA*, Bt03220821_m1), peroxisome proliferator activated receptor gamma (*PPARG*) (Bt03217547_m1), nuclear factor kappa B subunit 1 (*NFKB1*, Bt03243457_m1), thromboxane A2 receptor (*TBXA2R*, Bt04301659_m1), and beta-2-microglobulin (*B2M*, endogenous control, Bt03251630_g1). Genes were evaluated in triplicate with 2 × TaqMan Gene Expression Master Mix (Applied Biosystems), 20 × TaqMan Gene Expression Assay Mix (Applied Biosystems), sample cDNA (50 ng/well), and nuclease-free water for a total of 10 μL per reaction well. Thermal cycling conditions for the Fast 2-step PCR system were as follows: stage 1, 95°C for 20 s; stage 2, 95°C for 3 s; stage 3, 60°C for 30 s, with 40 cycles of stages 2 and 3. Data were recorded and compiled using ThermoFisher ExpressionSuite Software version 1.3.

### Statistical Analysis

Results for all analyses except for PCR are presented as the ratio of least squares means in treated cells to the untreated control + standard deviation. The results for PCR were analyzed with a modified ΔΔCt method and are graphically represented as 2^−Δ*ΔCt*^ (23). Sample size was calculated a priori based on unpublished preliminary apoptosis data. The PROC POWER statement was used in SAS 9.4 (Cary, North Carolina) with the following syntax: procpower; onewayanova; groupmeans = 1 (untreated media control), 1.11 (ethanol vehicle control), 1 (10 nM 15-F_2t_-IsoP), 18.6 (25 ng/mL LPS), 15.2 (25 ng/mL LPS + 10 nM 15-F_2t_-IsoP); stddev = 1.15; alpha = 0.05; npergroup =.; power = 0.9. The calculation suggested a sample size of at least 3 was sufficient to detect a difference of 1-fold change in apoptotic response. Data were analyzed for normality by visualizing Q-Q plots and confirmed with Shapiro-Wilk normality test. For commercial assays and PCR, a one-way ANOVA with Tukey's HSD posthoc test was performed. For analysis of endothelial cell barrier resistance, a two-way ANOVA was performed with the factors being time and treatment. Two-way ANOVA was followed by Tukey's HSD posthoc test. All statistical analyses were carried out with SAS 9.4.

## Results

### 15-F_2t_-IsoP Does Not Affect BAEC Viability During LPS Challenge

**Figure 2:**
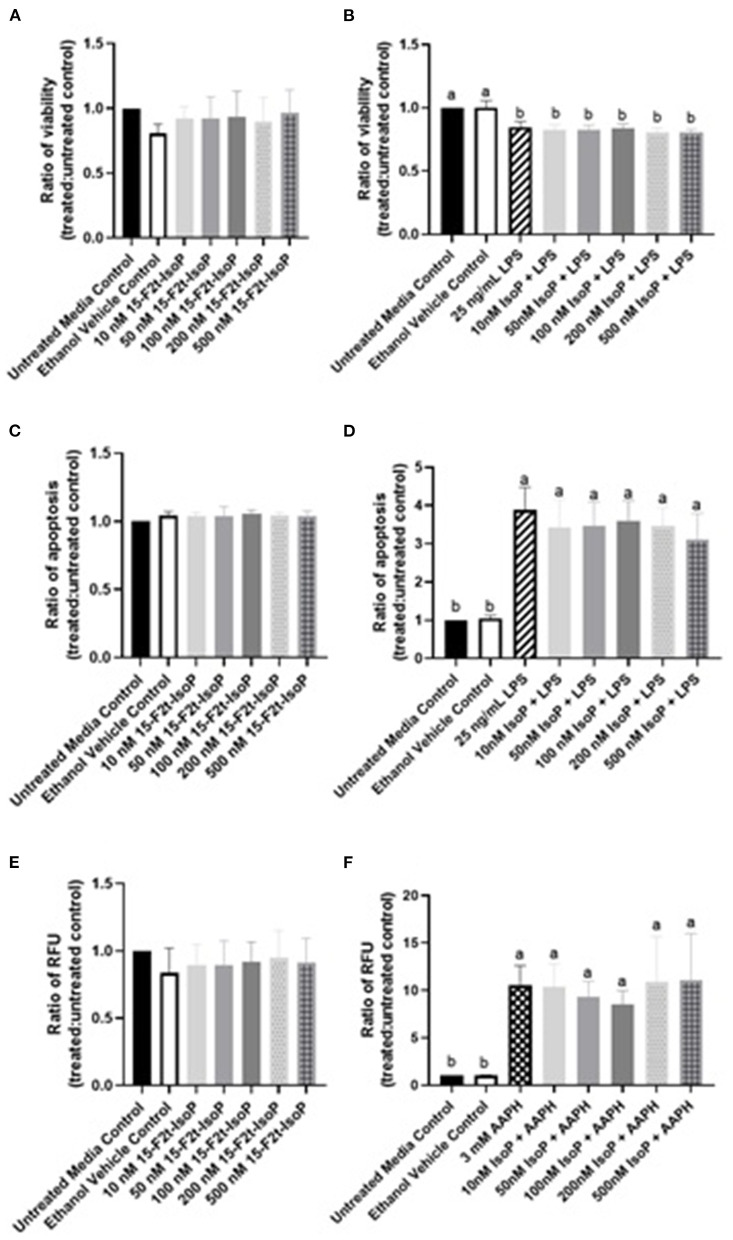
15-F_2t_-IsoP does not alter bovine aortic endothelial cell (*n* = 4) viability, apoptosis, or RONS production in either unstimulated [**(A)**, **(C)**, **(E)**, respectively] or stimulated cells after 12 h of incubation [**(B)**, **(D)**, **(F)**, respectively]. RFU, Relative fluorescence units. Different superscripts are different (*P* < 0.05).

Viability results at 4, 8, and 12 h were all similar and therefore, only 12 hr data are presented here. The viability of cells treated only with 10-500 nM 15-F_2t_-IsoP was not different from untreated cells (*P* = 0.64), suggesting that IsoP do not have a detrimental effect on BAEC viability (Figure 2A). In BAEC treated with LPS, viability was 85% of that seen in untreated cells (*P* < 0.0001). Furthermore, up to 500 nM 15-F_2t_-IsoP doses did not increase cell viability relative to LPS (*P* = 0.85; Figure 2B). Therefore, while 15-F_2t_-IsoP does not affect viability on its own, it also does not appear to reduce cell death during LPS challenge.

### 15-F_2t_-IsoP Does Not Alter Apoptosis in BAEC Under LPS Challenge

As above, 12 hr data were chosen to represent the effect of IsoP on apoptosis at all timepoints. Complimentary to our viability data, apoptosis in BAEC treated with only 15-F_2t_-IsoP was not altered from untreated controls (*P* = 0.64; Figure 2C). When cells were treated with LPS, apoptosis increased 389% after 12 hr compared to untreated cells (*P* < 0.0001). However, treating BAEC with LPS and 10-500 nM 15-F_2t_-IsoP did not decrease apoptosis relative to LPS only (*P* = 0.65; Figure 2D). Thus, 15-F_2t_-IsoP does not have an effect on BAEC apoptosis alone nor in the presence of LPS.

### 15-F_2t_-IsoP Does Not Change RONS Production in BAEC Under Oxidant Challenge

As with viability and apoptosis, only the 12 hr data are presented given 4, 8, and 12 hr results were similar. Compared to untreated controls, 15-F_2t_-IsoP did not alter RONS production on its own in BAEC (*P* = 0.87; Figure 2E). When challenged with the free radical generator AAPH, a 10-fold increase in BAEC RONS production was seen compared to media controls (*P* = 0.0015). When treated with 15-F_2t_-IsoP in addition to AAPH, RONS production was not different from AAPH alone (*P* = 0.99). However, RONS production was only 9-fold (*P*=0.007) and 8.5-fold (*P* = 0.02) higher than untreated cells at 50 and 100 nM doses of IsoP, respectively. These data therefore demonstrate a relative decrease of RONS production at intermediate doses (Figure 2F).

### 15-F_2t_-IsoP Does Not Affect BAEC Barrier Integrity During LPS Challenge

**Figure 3:**
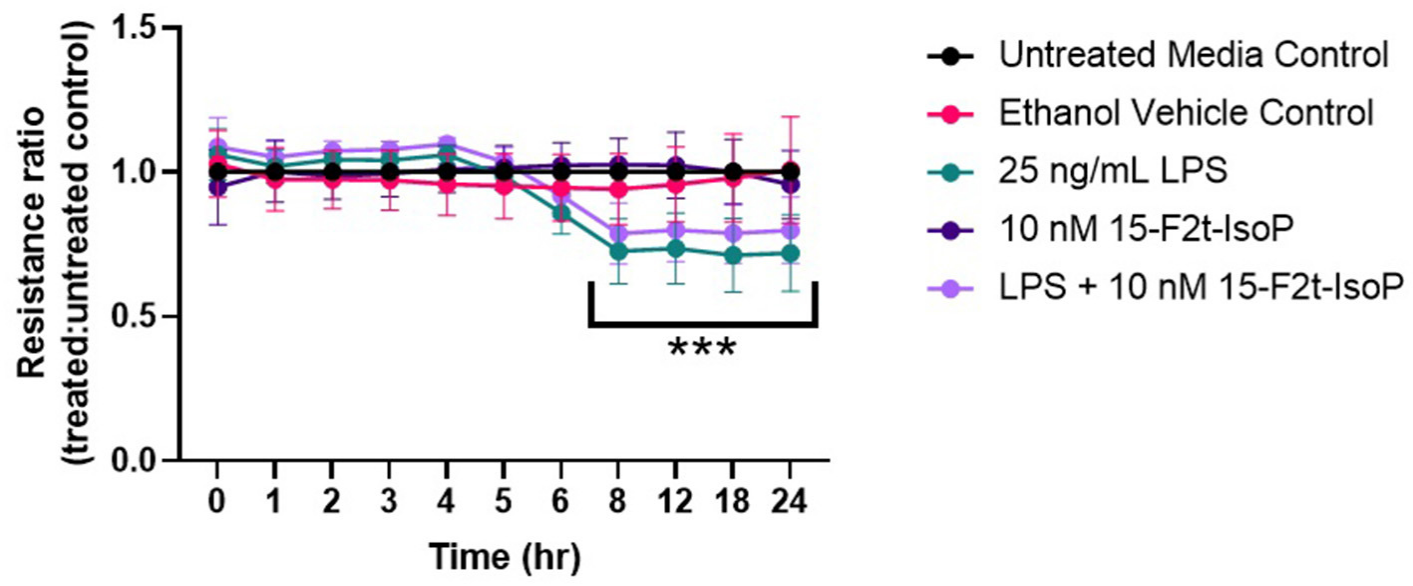
Bovine aortic endothelial cell (*n* = 4) barrier integrity is not significantly improved by 15-F_2t_-IsoP during lipopolysaccharide (LPS) challenge. *Significant time x treatment interaction (*P* < 0. 05).

As maintaining endothelial cell barrier integrity is a critical component of appropriate inflammatory responses, we were interested in assessing if IsoP could improve the resistance across a BAEC monolayer under LPS challenge. A significant time-treatment interaction was detected (*P* < 0.0001; Figure 3). Between 8 and 24 hr, barrier integrity was significantly decreased in LPS-treated cells compared to the untreated media control (*P* < 0.0001). A relative increase of barrier integrity was noted in LPS + 15-F_2t_-IsoP-treated BAEC compared to LPS only between 8 and 24 hr (*P* = 0.06).

### Inflammatory Gene Expression Is Not Altered by 15-F_2t_-IsoP

**Figure 4:**
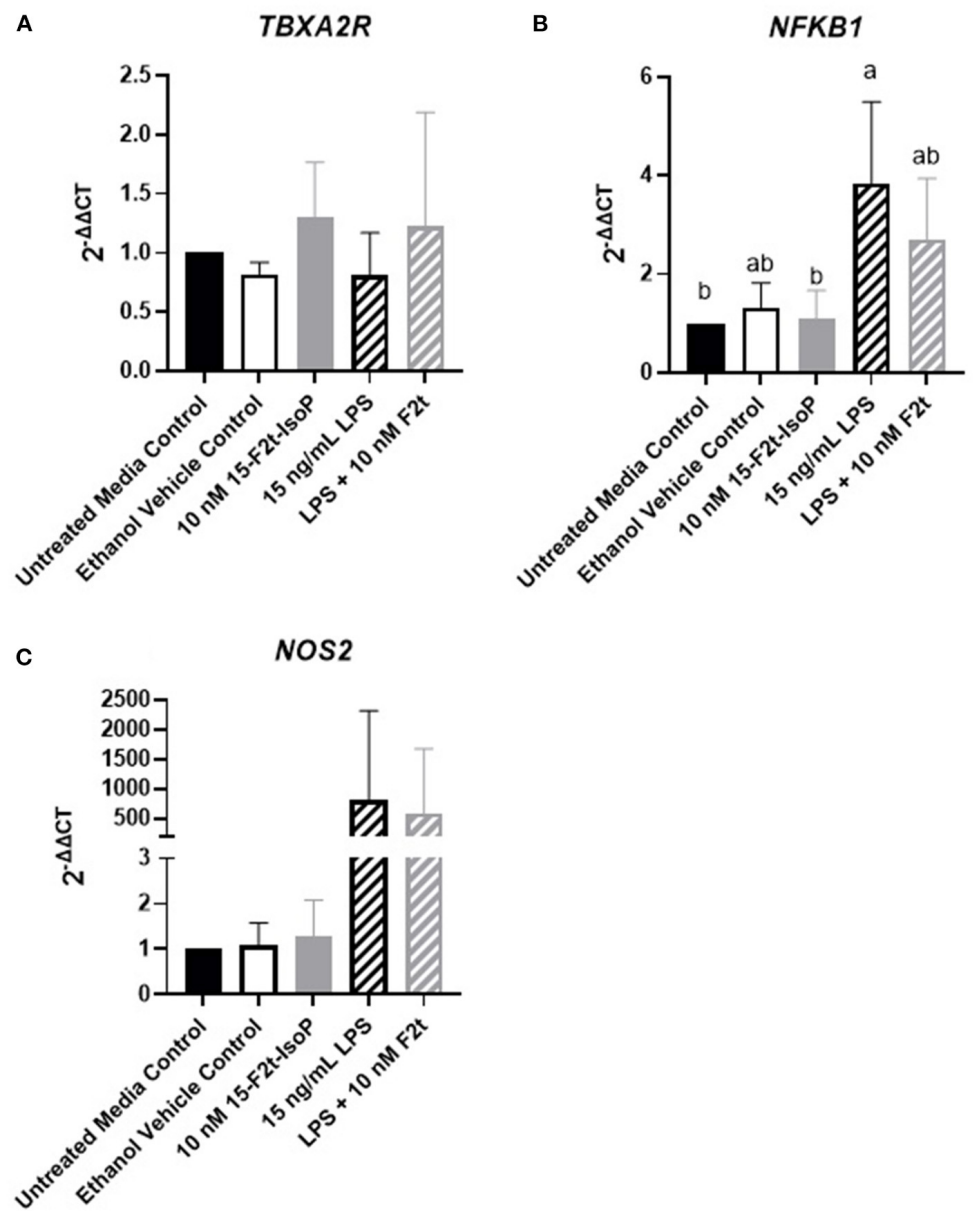
Gene expression of relevant inflammatory genes in bovine aortic endothelial cells (*n* = 4) treated with lipopolysaccharide (LPS), 15-F_2t_-IsoP, or both for 4 h. **(A)** Thromboxane A2 receptor (*TBXA2R*). **(B)** Nuclear factor kappa B subunit 1 (*NFKB1*). **(C)** Nitric oxide synthase 2 (*NOS2*). Different superscripts are different (*P* < 0.05).

One possible mechanism involved with the relative increase in barrier integrity described above could be due to alterations in inflammatory gene regulation. In relevant inflammatory genes, IsoP did not have an appreciable effect on the gene expression of BAEC challenged with LPS. As the data were similar across genes and timepoints, *TBXA2R, NFKB1*, and *NOS2* at 4 hr were selected as representatives to be reported herein (Figures 4A–C, respectively). Table 1 shows the *P* values for all genes and timepoints assessed. There was a higher expression of *NFKB1* and *NOS2* in LPS-treated cells compared to those without the agonist (*P* = 0.001 and 0.002, respectively). However, no differences were noted between LPS and LPS + 15-F_2t_-IsoP groups (*P* > 0.05). Therefore, IsoP did not influence inflammatory gene expression in the face of LPS challenge.

**Table 1:** *P*-values for each inflammatory gene assessed at 4-, 8-, and 12-h treatment incubations.

	**4 h**	**8 h**	**12 h**
Thromboxane A2 receptor (*TBXA2R*)	0.9	0.97	0.92
Nuclear factor kappa B subunit 1 (*NFKB1*)	0.001	0.0001	0.0001
Peroxisome proliferator activated receptor alpha (*PPARA*)	0.2	0.45	0.95
Peroxisome proliferator activated receptor gamma (*PPARG*)	0.29	0.86	0.18
Nitric oxide synthase 2 (*NOS2*)	0.002*	0.002	0.0001

*Overall ANOVA value was significant, but no differences were detected between treatment groups after adjusting for multiple comparisons.

**Figure 5:**
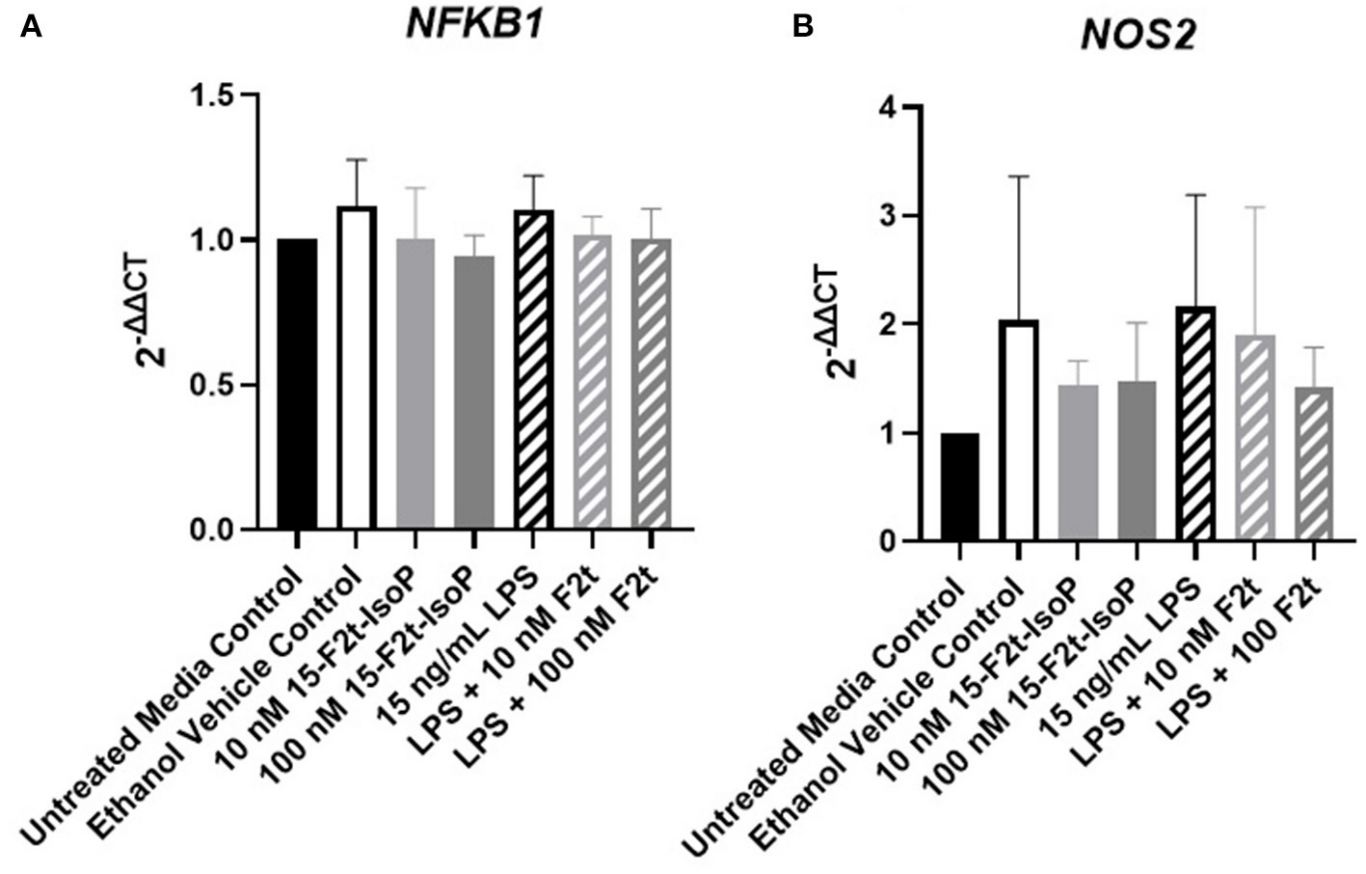
Gene expression of nuclear factor kappa B subunit 1 [*NFKB1*; **(A)**] and nitric oxide synthase 2 [*NOS2*; **(B)**] in bovine aortic endothelial cells (*n* = 4) treated with lipopolysaccharide (LPS), 15-F_2t_-IsoP, or both for 1 h.

To ensure the lack of change in inflammatory gene expression was not due to prolonged time in cell culture or low dosing of IsoP, we also evaluated *TBXA2R, NFKB1*, and *NOS2* expression at 1 hr with 10 and 100 nM IsoP concentrations. Similar to the 4, 8, and 12 hr timepoints, no differences were noted between any treatment group for any of the genes (*P* > 0.14). However, numerical decreases in *NFKB1* (Figure 5A) and *NOS2* (Figure 5B) expression were demonstrated in LPS and 15-F_2t_-IsoP compared to LPS treatments (*P* = 0.64 and 0.52, respectively). Moreover, although a relative increase of *NOS2* expression was seen in ethanol-treated cells compared to untreated controls at 1 hr, the IsoP treatments that were delivered with the vehicle did not appear to be affected to a similar degree (*P* = 0.63).

## Discussion

The data presented herein demonstrate that 15-F_2t_-IsoP does not alter viability, apoptosis, barrier integrity, or gene transcription networks of acute inflammation and EC dysfunction in our *in vitro* BAEC inflammation model. However, there was evidence of cytoprotection during the agonist challenge. This study is important for bovine medicine and dairy science because it clarifies effects IsoP do not have during acute EC inflammation characteristic of bovine mastitis.

As oxylipids are potent inflammatory mediators, it stands to reason that IsoP are capable of participating in mastitis pathophysiology. Decreased EC viability and increased apoptosis are common outcomes resulting from dysregulated inflammation and oxidative stress (24). Past evidence suggested that IsoP affect viability in EC. For example, Yura et al. (25) detailed that 15-F_2t_-IsoP increased viability in BAEC at concentrations similar to those used at present. Conversely, Brault and colleagues established that 15-F_2t_-IsoP decreased brain microvascular endothelial cell viability and did not affect viability of human umbilical vein endothelial cells. Evidence of IsoP altering apoptosis is scarce in the literature. In our model, however, IsoP did not affect viability or apoptosis. One plausible explanation for the differences between our results and those reported previously may be due to differences in methodology. Brault, Martinez-Bermudez (26), for example, measured viability with MTT assays as opposed to the ATP-based assay we used. Furthermore, ample evidence suggests that the action of IsoP depends on the vascular bed being studied (27). Therefore, BAEC viability and apoptosis may not be affected while EC from other locations (e.g., mammary EC) might. Besides mammary EC, inflammatory regulation in the mammary gland during mastitis involves many cell types and mediators, which can be challenging to replicate *in vitro* with a single-cell model (28). Further research is needed to determine the effect of IsoP in specific tissue microenvironments.

Increased RONS production can also participate in mastitis pathophysiology. Unopposed excessive RONS directly contribute to oxidative stress by causing damage to host DNA, proteins, and lipids. Once macromolecules are damaged, normal cellular function is impaired (2). For instance, stimulating bovine mammary epithelial cells with hydrogen peroxide resulted in substantial lipid peroxidation, altered cell morphology, decreased cell proliferation, decreased antioxidant activity, and decreased viability (29). Furthermore, the lipid peroxidation products formed during oxidative stress can further perpetuate macromolecule damage (30, 31). Thus, we were interested in determining if IsoP could affect RONS production given their mechanism of formation. While RONS production was not similar to amounts seen in untreated controls presently, 50 and 100 nM 15-F_2t_-IsoP combined with AAPH showed a numerical decrease from cells treated only with AAPH. In contrast to our data, electrophilic arachidonic acid-derived IsoP (e.g., 15-A_2_/J_2_-IsoP) can increase ROS production *via* depletion of the antioxidant glutathione in neurons (31). However, 15-F_2t_-IsoP lacks the cyclopentenone moiety that is attributed to electrophilic IsoP reactivity. Therefore, how 15-F_2t_-IsoP might cause an absolute decrease in RONS production in a mastitis model remains unclear but warrants further investigation.

Classical signs of clinical mastitis, such as heat and swelling of the mammary gland, can be attributed to decreased EC barrier integrity (8). We observed a relative increase in barrier integrity of BAEC treated with LPS and 15-F_2t_-IsoP compared to LPS alone. This cytoprotective effect was interesting considering omega-6-derived oxylipids are commonly associated with negative impacts on barrier integrity. In fact, enzymatically-derived 13-HPODE and 20-HETE decreased barrier integrity in bovine EC (7, 8). Furthermore, Hart, Karman (32) found 500 nM 15-F_2t_-IsoP decreased barrier integrity in pulmonary artery EC. Some oxidized phospholipid products may be beneficial to the EC barrier, however. Birukov, Bochkov (33) detailed a barrier-protective effect of the epoxyisoprostane-containing phospholipid, 1-palmitoyl-2-(epoxyisoprostane E2)-sn-glycero-3-phosphocholine. Thus, certain IsoP may be capable of maintaining EC barrier integrity. Future studies should investigate how various IsoP isomers impact monolayer barrier integrity to determine which would be most beneficial to dairy cattle during mastitis.

Many inflammatory gene networks are altered during mastitis. For example, cells stimulated by LPS, as would be the case during coliform mastitis, show increased *NOS2* and *NFKB1* expression (34, 35). Activation of *NFKB1* suppresses the activity of *PPARA* and *PPARG* (36, 37). An additional gene important for EC inflammatory responses is *TBXA2R*, of which IsoP serve as a ligand (38). Previous studies have demonstrated that various IsoP can modify the aforementioned genes. Brooks, Musiek (21) found that the omega-3-derived 15-A_3t_-IsoP inhibited *NFKB1* in macrophages. Moreover, Bosviel, Joumard-Cubizolles (20) showed activation of *PPARG* in macrophages treated with omega-3-derived IsoP. Although previous studies have shown an effect, we did not observe a change in the gene expression as a result of IsoP treatment. However, it was intriguing to see a more pronounced, albeit non-significant, alteration in gene expression when cells were treated for 1 hr compared to 4, 8, or 12 hr. Given their short half-life, these results support that IsoP may not be stable in cell culture for extended periods of time (39). Further characterization of optimal dosing and treatment timing should serve as the basis for future studies.

## Conclusions

In our BAEC inflammatory model, IsoP do not alter viability, apoptosis, reactive metabolite production, barrier integrity, or gene transcription networks. However, time of exposure and doses may be important factors that can be regulated by the tissue environment and therefore are complex to model *in vitro*. Advanced modeling including cellular bioenergetics may be necessary to realize a significant cytoprotective effect of IsoP in bovine cells and should be the goal of future studies.

## Data Availability Statement

The original contributions presented in the study are included in the article/supplementary material, further inquiries can be directed to the corresponding author.

## Author Contributions

AP drafted the manuscript while GC provided suggestions on editing. AP and GC reviewed and approved the submitted manuscript. All authors participated in the conception of this study and contributed equally to interpreting the results. All authors contributed to the article and approved the submitted version.

## Funding

This project was completed with funding support from the following entities: Agriculture and Food Research Initiative (AFRI) Competitive Grants Program (2014-68004-21972, 2017-67015-26676, and 2017-38420-26759) from the USDA National Institute of Food and Agriculture, AFRI Predoctoral Fellowship Program (2022-67011-36561) from the USDA National Institute of Food and Agriculture, an endowment from the Matilda R. Wilson Fund (Detroit, MI, USA), and the Michigan Alliance for Animal Agriculture.

## Conflict of Interest

The authors declare that the research was conducted in the absence of any commercial or financial relationships that could be construed as a potential conflict of interest.

## Publisher's Note

All claims expressed in this article are solely those of the authors and do not necessarily represent those of their affiliated organizations, or those of the publisher, the editors and the reviewers. Any product that may be evaluated in this article, or claim that may be made by its manufacturer, is not guaranteed or endorsed by the publisher.
